# Sex differences in atrial potential morphology

**DOI:** 10.1016/j.ijcha.2024.101597

**Published:** 2025-01-03

**Authors:** Danny Veen, Ziliang Ye, Mathijs S. van Schie, Paul Knops, Maryam Kavousi, Lara Vos, Vehpi Yildirim, Yannick J.H.J. Taverne, Natasja M.S de Groot

**Affiliations:** a Dept. of Cardiology, Erasmus University Medical Center, Rotterdam, the Netherlands; b Dept. of Epidemiology, Erasmus MC, University Medical Center, Rotterdam, the Netherlands; c Dept. of Cardio-Thoracic Surgery, Erasmus University Medical Center, Rotterdam, the Netherlands; d Dept. of Micro-electronics, Circuits and Systems, Faculty of Electrical Engineering, mathematics and computer sciences, Delft University of Technology, Delft, the Netherlands

**Keywords:** Sex differences, Atrial mapping, Conduction disorders, Potential morphologies

## Abstract

**Background:**

Areas of conduction disorders play an important role in both initiation and perpetuation of AF and can be recognized by specific changes in unipolar potential morphology. For example, EGM fractionation may be caused by asynchronous activation of adjacent cardiomyocytes because of structural barriers such as fibrotic strands. However, it is unknown whether there are sex differences in unipolar potential morphology. Therefore, atrial potential morphologies during sinus rhythm (SR) were compared between male and female patients.

**Methods:**

Based on propensity score matching, 62 male and female patients in whom high-resolution mapping of the right atrium (RA), left atrium (LA), and pulmonary vein area (PVA) including Bachmann’s bundle (BB) was performed during coronary bypass grafting surgery and/or valvular heart surgery. Unipolar potentials were classified as single potentials (SPs), short double potentials (SDPs), long double potentials (LDP), fractionated potentials (FPs) and fraction duration (FD). The proportion of conduction block lines was also determined.

**Results:**

Female patients had a higher proportion of SDPs, LDPs and FPs at the RA, and SDPs at BB. At the PVA, there were less SPs and more SDPs and FPs. In females, FDs were longer at the RA and PVA, and potential voltages of only SPs were lower at the RA (all P < 0.05). Females also had more CB at the RA and at PVA (P < 0.05).

**Conclusion:**

In females, the proportion of single unipolar potentials indicative of smooth conduction, was lower compared to males, at the RA and PVA and to a lesser degree at BB. Females also had more CB at RA and PVA. Hence, these results may reflect sex-differences in the degree of electrical remodeling.

## Highlights

• Sex differences in atrial potential morphology (during SR) of the atria between males and females were particularly found at the right atrium, Bachmann’s Bundle and to a lesser degree at the pulmonary vein area.
• In female patients, both the right atrium and Bachmann’s Bundle and the pulmonary vein area contained more short-double, long double, fractionated potentials and conduction block lines. Additionally, females had lower unipolar potential voltages.

## Graphical abstract

**Figure:**
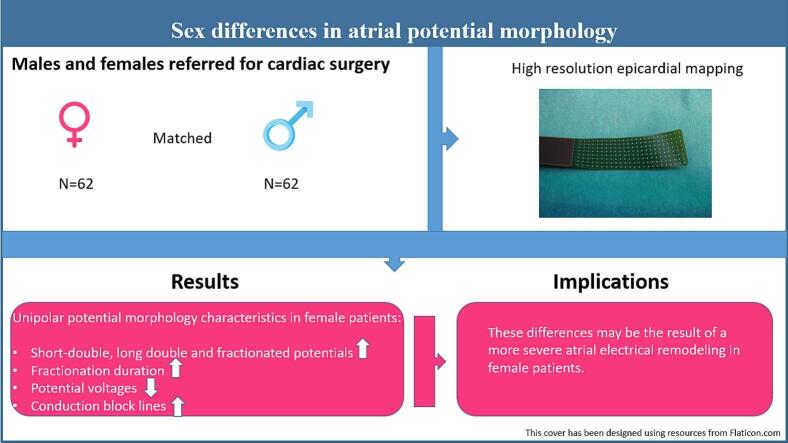

## 1 Introduction

Sex differences in the pathophysiology underlying atrial fibrillation (AF) can no longer be ignored as there are many reports on sex differences in AF epidemiology, clinical manifestation and treatment outcomes [1], [2], [3], [4], [5], [6], [7], [8], [9], [10]. Knowledge on sex differences in electrical atrial remodeling is, however, scarce. A recent intra-operative mapping study of both atria demonstrated that even during sinus rhythm (SR) females who did not have AF have more CB (conduction times (CTs) > 12 ms were defined as conduction block (CB)) and low-voltage areas, [8] Areas of conduction disorders play an important role in both initiation and perpetuation of AF and can be recognized by specific changes in unipolar potential morphology. Atrial potentials recorded from CB areas are mainly double potentials of which each deflection represents activation at either side of the CB line, or fractionated potentials indicating a wavefront which is turning around the end of CB lines.

Sex differences have been found in the proportion of fractionated fibrillation potentials, as electro-anatomical mapping of the left atrium in 116 patients (42 females) with episodes of paroxysmal or persistent AF prior to pulmonary vein isolation demonstrated that voltages of bipolar potentials were lower and that the proportion of fractionated potentials were higher in female patients. [11], [12] These observations suggest that the female atria are more vulnerable to remodeling. Whether these sex differences in potential morphology also exist during SR in patients without AF-induced remodeling is unknown.

Recently, Ye et al. [13], [14] introduced the Electrical Fingerprint Score as a novel diagnostic tool to quantify the severity of conduction disorders, solely using unipolar potential morphology. In the present study, we examined whether there were regional sex differences in atrial potential morphology as an indicator of the degree of electrical remodeling of the atria during SR in patients undergoing cardiac surgery.

## 2 Methods

### 2.1 Study population

The study population was retrieved from an existing database containing patients who had undergone a high-resolution mapping procedure during coronary artery bypass grafting. None of these patients had a history of AF. Baseline characteristics of the available male (N = 140) and female (N = 162) patients differed in underlying concomitant valvular heart disease, (P = 0.014)), diabetes-mellitus (P = 0.003) and dyslipidemia (P = 0.038). All females were selected and matched with males on these 3 variables (using the method of Optimal propensity-score matching based on a logistic regression model) resulting in a 1:1 female/male ratio. This study was part of two prospective observational projects including Quest for Arrhythmogenic Substrate of Atrial fibrRillation (QUASAR, MEC 2010–054) and Hsf1 Activators Lower cardio myocyte damage Towards a novel approach to REVERSE AF (HALT & REVERSE, MEC 2014–393). Patients who had prior ablation therapy of atrial tachyarrhythmias, severe renal failure, an atrial pacing device or who required inotropic support were excluded from this study. Both projects were approved by the local ethics committee of the Erasmus Medical Centre and adhered to the Declaration of Helsinki principles; written consent was obtained from participating patients before surgical intervention.

### 2.2 Intra-operative mapping procedure

As previously described, high-resolution mapping of the epicardium was performed before commencement to extra-corporal circulation. [14], [15], [16], [17] A bipolar pacemaker wire was stitched to the epicardial wall of the right atrial appendage and used a reference electrode, whereas a steel wire fixed to the subcutaneous tissue was used as an indifferent electrode. A 128- or 192 unipolar electrode array was used for mapping of the different atrial regions (inter-electrode distances of 2.0 mm). Using a predefined mapping scheme, the entire epicardial surface of the RA, BB, LA and PVA was covered. RA mapping started at the cavo-tricuspid isthmus towards the vena cava superior. Mapping of BB started from the tip of the left atrial appendage across the roof of the LA, behind the aorta towards the superior cavo-atrial junction and mapping of the LA from the lower border of the left inferior pulmonary vein (PV) along the left atrio-ventricular groove towards the left atrial appendage. The area between the pulmonary veins (PVA) was accessed from the oblique sinus. Five seconds of SR were collected at every mapping site. The recordings consisted of unipolar epicardial electrograms, a bipolar reference electrogram, a surface ECG lead and a calibration signal of 2 mV and 1000 ms. All data were stored on hard disk after amplification (gain 1000), filtering (bandwidth 0.5–400 Hz), sampling (1 KHz) and analogue-to-digital conversion (16 bits).

### 2.3 Mapping data analysis

**Fig. 1:**
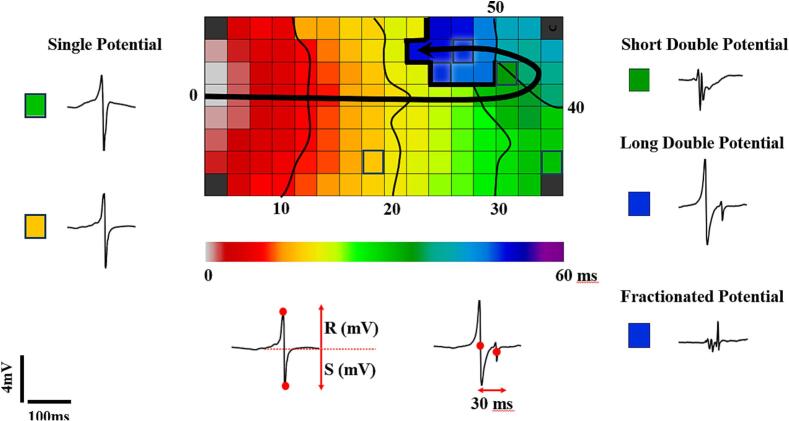
Color-coded local activation map obtained from the right atrium of a 65-year-old male patient with coronary artery disease and CB in the upper part of the mapping area which forces the wavefront to turn around this area, giving rise to long double potentials LDP and FP. Examples of calculations of the RS ratio and fractionation duration are shown in the lower panel. Isochrones are drawn at 10 ms intervals, the black arrow shows the main propagation trajectory of the sinus rhythm wavefront and the thick black indicates an area of conduction block. CB: conduction block, FP: fractionated potentials, LDP: long double potentials.

Custom-made software was used to automatically measure electrogram (EGM) characteristics. EGMs of poor quality or ectopic beats were excluded from analysis. The steepest negative slope of a unipolar potential was annotated to construct local activation time (LAT) maps. Unipolar potentials were classified into single potentials (SPs (single negative deflection)), short double (SDPs (interval between deflections < 15 ms)), long double (LDPs (deflection interval ≥ 15 ms)) or fractionated potentials (FPs (≥3 deflections, as illustrated in Fig. 1)). [13], [14] Fractionation duration (FD) was defined as the time difference (ms) between the first and last deflection of non-SPs. [13], [14] As described in our previous study, SPs were classified according to their differences in relative R- and S-wave amplitudes and scaled from −1 (R-wave) to 1 (S-wave). [15].

CB was defined as conduction times (CTs) > 12 ms, corresponding with a conduction velocity: <18 cm/s. The prevalence of CB lines is expressed as a percentage of the total available number of inter-electrode connections. The length of CB lines was measured on a 2 mm resolution scale as described before. [16], [17].

### 2.4 Statistical analysis

Statistical analysis was performed using IBM SPSS Statistics (version 28) and R Studio (version 4.2.2). The distribution of continuous variables was examined using the Shapiro–Wilk test. If normally distributed, the mean and standard deviation (SD) were used to describe continuous variables and the differences between groups were compared by using the independent sample T-test. Continuous variables that were not normally distributed were described by the median and interquartile range (IQR) whereas the differences between groups were compared using the Wilcoxon rank sum test. Categorical variables were described by the number and percentage, and the Chi square or McNemar test was used to compare differences.

## 3 Results

### 3.1 Study population

After matching, the dataset consisted of 62 male and 62 female patients. Characteristics of females (mean age: 68±9 years) and matched male patients (mean age: 66 ± 10 years) are summarized in Table 1. In both groups, most patients had a normal left ventricular function (females: 74 %, males: 76 %) and non-dilated atria (females: 85 %, males: 77 %).

**Table 1:** Baseline characteristics of females and matched males.

Variables	Female	Male	P-Value
N	62	62	
Age, year, mean (SD)	68 (9.24)	66 (9.70)	0.194
BMI, kg/m^2^, mean (SD)	28.63 (4.68)	27.84 (4.38)	0.337
Underlying heart diseases			0.699
CABG	41 (66.13)	44 (70.97)	
CABG + VHD	21 (33.87)	18 (29.03)	
Hypertension, N (%)	41 (66.13)	39 (62.90)	0.851
Dyslipidemia, N (%)	34 (54.84)	26 (41.94)	0.208
Diabetes mellitus, N (%)	32 (51.61)	33 (53.23)	1.000
Myocardial infarction, N (%)	22 (35.48)	22 (35.48)	0.603
Left ventricular function, N (%)			0.950
Normal (EF > 55 %)	46 (74.19)	47 (75.81)	
Mild impairment (EF 46 %–55 %)	10 (16.13)	10 (16.13)	
Moderate impairment (EF 36 %–45 %)	6 (9.68)	5 (8.06)	
Severe impairment (EF < 35 %)	−	−	
Left atrial dimension, N (%) (<45 mm /LAVI of < 35 ml/m^2^)	53 (86)	48 (77)	0.112
ACEI/ARB/AT2 antagonist, N (%)	9 (14.52)	14 (22.58)	0.706
Statin, N (%)	57 (91.94)	57 (91.94)	1.000
**Antiarrhythmics**			
Class I, N (%)	62 (100.00)	62 (100.00)	NA
Class II, N (%)	48 (77.42)	48 (77.42)	1.000
Class III, N (%)	0 (0.00)	1 (1.61)	1.000
Class IV, N (%)	4 (6.45)	5 (8.06)	1.000
Digoxin, N (%)	1 (1.61)	1 (1.61)	1.000
**Hormone replacement therapy**			
Estriol cream, N (%)	1 (6.2)	−	
Synapause cream, N (%)	1 (6.2)	−	

ACEI: angiotensin-converting enzyme inhibitors, ARB: angiotensin receptor blockers, AT2: angiotensin type 2 receptor, BMI: body mass index, CABG: coronary artery bypass grafting, EF: ejection fraction, N: number, SD: standard deviation, VHD: valvular heart disease, NA: not available.

### 3.2 Sex differences in unipolar potential morphology

A total of 1,172,199 atrial potentials were recorded (males: 579,462 (9,346 ± 2,860) potentials versus females: 592,737 (9,560 ± 2,809) potentials) during an average SR cycle length of 884 ± 174 ms (males: 844 ± 174 ms, females: 832 ± 165 ms, P = 0.086). Table 2 summarizes sex differences in potential morphology for every region separately.

**Table 2:** Regional sex differences in potential morphologies.

Potential Type and Location (median and IQR)	Female	Male	P- Value
N	62	62	
**All atrial potentials**			
SPs (%)	82.16 [77.54–85.94]	85.59 [83.83–88.42]	**0.001**
SDPs (%)	11.08 [9.29–3.41]	8.99 [7.60–10.42]	**0.001**
LDPs (%)	4.39 [2.54–7.01]	3.93 [2.19–4.97]	**0.027**
FPs (%)	1.75 [1.02–2.94]	0.98 [0.75–1.76]	**0.001**
FD (ms)	11.00 [9.00–13.75]	10.00 [9.00–12.00]	0.176
R/S ratio	0.45 [0.39–0.53]	0.45 [0.41–0.53]	0.881
CB (%)	2.38 [1.70–3.74]	2.01 [1.31–2.41]	**0.003**
			
**RA**			
SPs (%)	82.67 [77.07–88.89]	86.68 [82.94–91.16]	**0.001**
SDPs (%)	9.80 [6.34–11.20]	7.50 [5.04–9.97]	**0.042**
LDPs (%)	5.80 [3.26–9.07]	4.04 [1.24–6.47]	**0.003**
FPs (%)	1.64 [0.57–3.53]	0.94 [0.25–1.93]	**0.003**
FD (ms)	13.50 [10.00–16.00]	11.00 [8.00–14.00]	**0.008**
R/S ratio	0.59 [0.51–0.64]	0.57 [0.51–0.64]	0.379
CB (%)	3.37 [2.27–5.65]	2.30 [1.09–3.16]	**<0.001**
			
**BB**			
SPs (%)	78.75 [70.83–85.84]	84.91 [73.25–89.09]	0.063
SDPs (%)	13.67 [10.21–17.92]	10.74 [6.62–14.43]	**0.004**
LDPs (%)	3.98 [0.84–7.46]	3.15 [0.72–8.05]	0.986
FPs (%)	1.54 [0.73–3.25]	0.79 [0.17–2.85]	0.050
FD (ms)	10.00 [8.25–13.00]	11.00 [8.00–14.00]	0.337
R/S ratio	0.63 [0.56–0.73]	0.68 [0.62–0.76]	**0.046**
CB (%)	2.79 [1.42–5.07]	2.65 [1.02–5.46]	0.557
			
**LA**			
SPs (%)	86.71 [74.14–90.59]	86.64 [82.14–89.94]	0.237
SDPs (%)	10.84 [7.15–16.55]	9.56 [6.70–11.51]	0.093
LDPs (%)	1.60 [0.26–6.33]	1.15 [0.31–3.46]	0.638
FPs (%)	0.72 [0.23–1.78]	0.70 [0.21–1.64]	0.513
FD (ms)	8.00 [7.00–13.00]	9.00 [7.00–11.00]	0.588
R/S ratio	0.05 [-0.15–0.19]	0.10 [-0.06–0.23]	0.176
CB (%)	0.74 [0.13–1.88]	0.68 [0.12–1.38]	0.385
			
**PVA**			
SPs (%)	82.91 [76.56–89.47]	88.74 [85.96–91.63]	**0.003**
SDPs (%)	12.77 [6.86–18.55]	9.08 [6.48–11.48]	**0.011**
LDPs (%)	1.81 [0.45–4.11]	0.83 [0.29–2.83]	0.181
FPs (%)	0.69 [0.21–2.72]	0.39 [0.12–1.14]	**0.037**
FD (ms)	17.09 [10.53–23.44]	11.26 [8.37–14.04]	**0.003**
R/S ratio	0.29 [0.11–0.42]	0.26 [0.06–0.44]	0.582
CB (%)	3.35 [2.40–4.77]	2.29 [1.58–3.56]	**0.010**
			
**Atrial potential voltages**			
SPs (mV)	4.73 [3.68–5.69]	5.13[4.42–6.14]	0.206
SDPs (mV)	3.07 [2.36–3.54]	3.04 [2.50–3.89]	0.468
LDPs (mV)	1.61 [1.14–2.17]	1.84 [1.35–2.65]	0.156
FPs (mV)	1.76 [1.27–2.46]	1.76 [1.18–2.16]	0.702
			
**RA**			
SPs (mV)	4.46 [3.69–5.77]	4.98 [4.33–6.18]	**0.043**
SDPs (mV)	2.30 [1.79–2.86]	2.72 [2.07–3.07]	0.053
LDPs (mV)	1.34 [1.05–1.69]	1.43 [1.04–1.93]	0.554
FPs (mV)	1.25 [0.92–1.65]	1.52 [0.99–1.98]	0.092
			
**BB**			
SPs (mV)	5.33 [3.53–6.24]	5.99 [3.95–7.34]	0.104
SDPs (mV)	2.97 [2.23–3.90]	3.28 [2.16–4.18]	0.508
LDPs (mV)	2.41 [1.32–3.43]	2.09 [1.12–3.70]	0.470
FPs (mV)	2.10 [1.24–2.98]	2.13 [1.36–2.96]	0.668
			
**LA**			
SPs (mV)	5.12 [2.77–7.63]	6.37 [3.49–7.83]	0.208
SDPs (mV)	3.98 [3.01–4.76]	4.19 [3.09–5.08]	0.462
LDPs (mV)	3.24 [1.66–5.27]	4.07 [2.63–5.71]	0.172
FPs (mV)	2.64 [2.04–3.70]	2.92 [2.24–3.91]	0.239
			
**PVA**			
SPs (mV)	4.77 [2.70–7.09]	4.06 [2.38–6.55]	0.459
SDPs (mV)	3.33 [2.20–4.76]	2.60 [1.64–4.94]	0.419
LDPs (mV)	2.18 [1.43–3.47]	1.43 [1.06–2.85]	0.124
FPs (mV)	2.77 [1.51–3.88]	2.18 [1.32–4.07]	0.440
			
**Cycle length (mean, SD)**	832 ± 165	884 ± 174	0.086
**Number of potentials per patient (mean, SD)**	9.560 ± 2809	9.346 ± 2860	0.711
**Total**	592.737	579.462	
**RA**	268.816	262.851	
**BB**	63.941	69.609	
**PV**	141.737	129.599	
**LA**	118.243	117.403	

BB: Bachmann’s Bundle, CB: conduction block, LA: left atrium, PVA: pulmonary vein area, RA: right atrium, SD: standard deviation, SPs: single potentials, SDPs: short double potentials, LDPs: long double potentials, FD: fractionation duration, FPs: fractionated potentials, IQR: interquartile range.

**Fig. 2:**
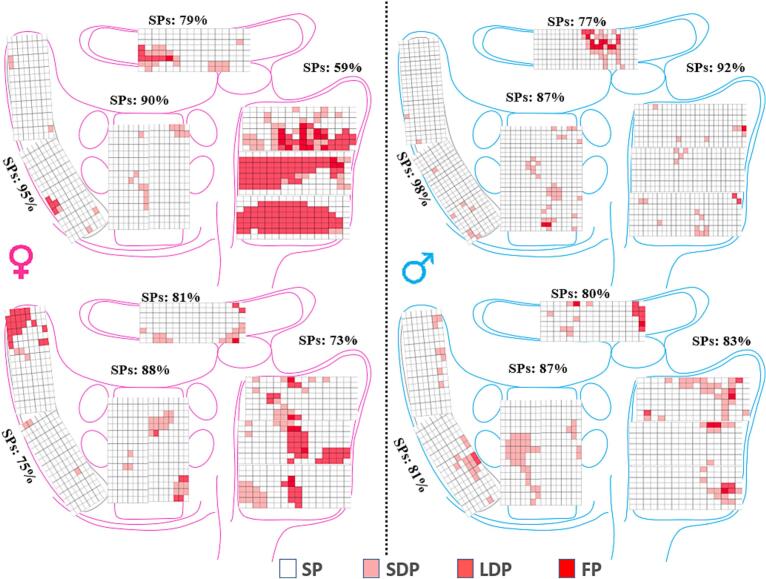
Representative examples of color-coded signal maps obtained from 2 female and male patients demonstrating from which electrode SPs, SDPs, LDPs and FPs are recorded. FPs: fractionated potentials, LDPs: long double potentials, SDPs: short double potentials, SPs: single potentials.

At the RA, the number of SPs was lower in female patients whereas the prevalence of SDPs, LDPs and FPs was higher in female patients (all P < 0.05). Sex differences in potential morphology at BB were limited to a higher number of SDPs in female patients (P = 0.004). At the LA, there were no differences in potential morphologies between males and females (all P > 0.05). Within the PVA, the number of SPs were lower in females and was associated with a higher number of SDPs and FPs (all P < 0.05). FD of FPs were longer in females only at the RA and PVA (all P < 0.05). (Fig. 2).

### 3.3 Sex differences in potential voltages

**Fig. 3:**
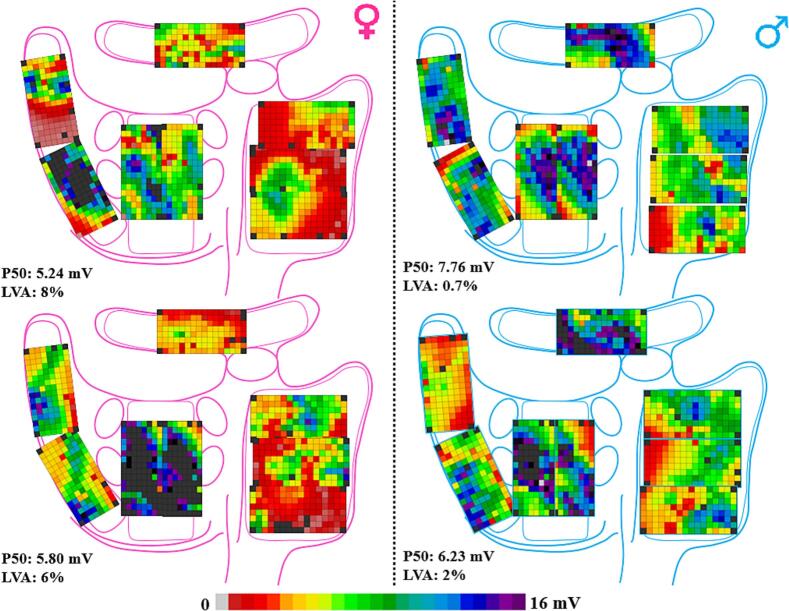
Representative examples of potential voltage maps obtained from 2 female and male patients demonstrating the median unipolar potential voltages and the percentage of low voltage areas. LVA: low voltage area.

Only at the RA, females had slightly lower SPs voltages; (4.46 [3.69–5.77] mV versus 4.98 [4.33–6.18] mV, P = 0.043 in males, Table 2). At all other sites, there were no significant sex differences in voltages of any potential type. (Fig. 3).

As can be seen in Table 2, there were no significant sex differences in R/S ratios of SPs at the RA, LA and PVA. Only at BB, R/S ratios differed slightly between females (0.63 [0.56–0.73]) and males (0.68 [0.62–0.76]) P = 0.046).

### 3.4 Sex differences in conduction block

Compared to males, the percentage of CB was significantly higher in female patients at both the RA (3.37 [2.27–5.65] % versus 2.30 [1.09–3.16] %, p < 0.001) and PVA (3,35 [2], [4]% versus 2,29 [1,58–3,56]%, p < 0,010) respectively). Nonetheless, at BB and LA, males and females had a similar proportion of CB (both P > 0.05).

## 4 Discussion

### 4.1 Key findings

*Key findings*.

In patients undergoing open heart surgery, sex differences in unipolar potential morphology during SR consist at all atrial sites with exception of the LA outside the PVA. In females, the proportion of single unipolar potentials indicative of smooth conduction, was lower compared to matched males, particularly at the RA and PVA and to a lesser degree at BB. FD of FPs were longer in females only at the RA and PVA and potential voltages were lower only at the RA. Females also had more CB at the RA and PVA.

### 4.2 Sex differences in unipolar potential morphology

EGM fractionation during SR is caused by local asynchronous activation of tissue beneath the recording electrodes. This can be related to characteristics of atrial tissue such as tissue anisotropy or local source-to-sink mismatches caused by branching of myocardial bundles. However, it can also be related to structural heterogeneity of cardiac tissue, due to e.g. deposition of fibrotic tissue. Subsequently slowing of conduction, conduction block and turning of wavefronts produce low voltage potentials, LDPs and FPs. In our study, female patients had more LDPs and FPs compared to males, suggesting that structural remodeling is more pronounced compared to matched males.

SDPs were more frequently recorded at BB in our female patients. Konings et al. performed intra-operative high density mapping studies in patients with the Wollf-Parkinson-White syndrome and found SDPs mainly along collisions sites. [18]. Simultaneous *endo*-epicardial mapping of the right atrial wall subsequently showed that SDPs may also be caused by a slight degree of electrical asynchrony across the atrial wall which in turn can also be related to structural remodeling. [17] Hence, the higher prevalence of SDP in female patients may reflect more extensive remodeling of BB.

### 4.3 Sex differences in structural remodeling

RA anatomy investigated by cardiac magnetic resonance images from formalin-preserved human hearts (N = 100, 48 ± 13 years, 55 % females) showed that there were no sex differences in RA anatomy. [19] The observed electrophysiological properties between male and female patients are more likely the result of different degrees of structural remodeling. There are indications that estrogen plays an important role in attenuation of remodeling [20]. In post-menopausal females, enhanced structural remodeling may be caused by upregulation of the expression of fibrosis related genes. [21] It has also been demonstrated that females have higher adipokine levels resulting in more inflammation and a higher degree of fibrotic remodeling in the atria as compared to males [22]. A higher degree of remodeling may result in lower unipolar potential voltages. Hence, the observed lower RA unipolar potential voltages in females compared to males may be a marker of increased structural remodeling in post-menopausal female patients. Indeed, in the present study, females had a lower proportion of SPs compared to males at the RA and BB, indicating less smooth propagation of the SR wavefront at these areas. This observation corresponds to prior findings that conduction disorders are more pronounced in female patients, particularly at the RA and to a lesser degree at BB. [8].

Prior studies demonstrated that left atrial dilatation is independently associated with female sex. In addition, the LA of females with AF contain a higher degree of fibrosis. [22] In our study population, consisting of female patients without AF, we detected sex differences in the left atrium only at the mapping area in between the pulmonary veins. These differences were limited to a decrease in the proportion of SPs, which indicates that there were less wavefronts propagating without encountering areas of conduction delay or block. This observation also supports the presence of enhanced structural remodeling in females compared to males.

## 5 Conclusion

Atrial unipolar potential morphologies differ between males and females at the RA, BB and to a lesser degree at PVA. Sex-differences in potential morphologies may reflect sex-differences in the degree of potential electrical remodeling, which can already be observed during SR in patients without atrial arrhythmias. As we performed intra-operative mapping, we only included patients who had an indication for cardiac surgery. Our findings can therefore not be translated to healthy subjects.

## 6 Clinical Implications

Even though the age-adjusted AF prevalence and incidence is higher in male patients, female patients have more frequently AF recurrences, more non-PV foci and a higher AF burden after PV isolation [7]. As electrical abnormalities during SR are already more pronounced in female patients, it may reflect a higher degree of structural, and hence electrical remodeling. Sex differences in remodeling may offer an explanation why the AF burden is higher and PV isolation is less successful in females.

## 7 Strengths and limitations

Our mapping study not only provides data on conduction inhomogeneity of the LA, but additionally also of PVA, BB and RA, in a population who does not have AF yet. As we performed intra-operative mapping, we only included patients who had an indication for cardiac surgery and thus severe cardiovascular disorders. Hence, our results cannot be simply extrapolated to other populations.

**Author contributions:**

Danny Veen: conceptualization, data analysis, writing and editing (this author takes responsibility for all aspects of the reliability and freedom from bias of the data presented and their discussed interpretation). **Shared first author**.

Ziliang Ye: data analysis and reviewing **Shared first author**.

Mathijs van Schie: data curation and reviewing.

Paul Knops: reviewing.

Maryam Kavousi: consultation on sex differences in cardiology.

Lara Vos: reviewing.

Vehpi Yildirim, PhD: reviewing.

Yannick Taverne: mapping during cardiac surgery and reviewing.

Natasja M.S. Groot, de: conceptualization, supervision, reviewing, editing and statistical support (this author takes responsibility for all aspects of the reliability and freedom from bias of the data presented and their discussed interpretation).

## 8 Sources of funding

We did not receive funding for completion of this manuscript.

## 9 Registration number of clinical studies

This study was performed as part of two prospective observational projects (MEC 2010–054 and MEC 2014–393) [10], [11], [12]. MEC 2010–054 and MEC 2014–393). Approval of both projects were granted by the local ethics committee of the 10.13039/501100010790Erasmus Medical Centre and adhere to the Declaration of Helsinki principles; written consent was obtained from participating patients before the surgical intervention.

## CRediT authorship contribution statement

**Danny Veen:** Writing – original draft, Methodology, Formal analysis. **Ziliang Ye:** Software, Formal analysis, Data curation. **Mathijs S. van Schie:** Writing – review & editing, Resources. **Paul Knops:** Writing – review & editing. **Maryam Kavousi:** Writing – review & editing. **Lara Vos:** Writing – review & editing. **Vehpi Yildirim:** Writing – review & editing, Project administration. **Yannick J.H.J. Taverne:** Writing – review & editing. **Natasja M.S de Groot:** Writing – review & editing, Supervision, Conceptualization.

## Declaration of competing interest

The authors declare that they have no known competing financial interests or personal relationships that could have appeared to influence the work reported in this paper.
